# Supplementary material for: A Propensity-Matched Retrospective Comparative Study with Historical Control to Determine the Real-World Effectiveness of Durvalumab after Concurrent Chemoradiotherapy in Unresectable Stage III Non-Small Cell Lung Cancer
Source: Cancers (Basel). 2023 Mar 5;15(5):1606. doi: 10.3390/cancers15051606 (PMC10000649; doi:10.3390/cancers15051606)
Supplement: Supplementary file 1 [file cancers-15-01606-s001.zip › Supplemetary tables.pdf]

Supplementary Table S1. Post-treatment progression pattern and subsequent treatment in patients with CCRT alone and durvalumab consolidation (DC).

Variables	Total (*n*=162)	CCRT Alone (*n*=119)	DC (*n*=43)	*p*-Value
Progression type				
Localized/Regional	68 (42.0)	52 (43.7)	16 (37.2)	0.223
Distant	74 (45.7)	50 (42.0)	24 (55.8)	
Unknown or Death	20 (12.3)	17 (14.3)	3 (7.0)	
Progression site				-
Lung	85 (52.5)	62 (52.1)	23 (53.5)	0.876
Lymph node	59 (36.4)	44 (37.0)	15 (34.9)	0.807
Pleura	15 (9.3)	12 (10.1)	3 (7.0)	0.761
Pericardium	4 (2.5)	3 (2.5)	1 (2.3)	1.000
Brain	26 (16.0)	18 (15.1)	8 (18.6)	0.594
Bone	14 (8.6)	10 (8.4)	4 (9.3)	1.000
Kidney	3 (1.9)	0 (0.0)	3 (7.0)	0.018
Adrenal	7 (4.3)	5 (4.2)	2 (4.7)	1.000
Liver	12 (7.4)	9 (7.6)	3 (7.0)	1.000
Spleen	1 (0.6)	0 (0.0)	1 (2.3)	0.265
Pancreas	2 (1.2)	1 (0.8)	1 (2.3)	0.462
Peritoneum	3 (1.9)	3 (2.5)	0 (0.0)	0.566
Unknown	7 (4.3)	5 (4.2)	2 (4.7)	1.000
Post-PD treatment				0.010
Local: OP, RT	39 (24.1)	29 (24.4)	10 (23.3)	
Systemic: CRT, Chemotherapy, TKI, ICI	82 (50.6)	53 (44.5)	29 (67.4)	
BSC or loss of follow up	41 (25.3)	37 (31.1)	4 (9.3)	

Values are presented as numbers (%). CCRT: concurrent chemoradiotherapy; PD: progressive disease; OP: operation; RT: radiotherapy; CRT: chemoradiotherapy; TKI: tyrosine kinase inhibitor; ICI: immune checkpoint inhibitor; BSC: best supportive care.

Supplementary Table S2. Multivariable Cox regression analysis for PFS and OS.

Variables	PFS		OS	
	**HR**	**95% CI**	**HR**	**95% CI**
DC	0.631	0.416 - 0.957	0.469	0.270 - 0.815
Anemia	1.214	0.779 - 1.891	0.818	0.475 - 1.407
Thrombocytopenia	0.659	0.249 - 1.741	0.474	0.104 - 2.153
Liver failure	0.808	0.351 - 1.858	0.649	0.191 - 2.210
BMI (18.5 – 25)	Ref	Ref	Ref	Ref
BMI < 18.5	1.041	0.485 - 2.236	1.128	0.452 - 2.814
25 ≤ BMI	0.740	0.441 - 1.241	0.670	0.355 - 1.264
eGFR (≥90)	Ref	Ref	Ref	Ref
0≤eGFR <60	1.313	0.505 - 3.419	1.387	0.480 - 4.008
60≤eGFR<90	1.045	0.658 - 1.658	1.235	0.694 - 2.200
Smoking (never)	Ref	Ref	Ref	Ref
Current smoker	1.155	0.509 - 2.618	1.803	0.529 - 6.150
Ex-smoker	1.339	0.615 - 2.916	1.555	0.468 - 5.168
ECOG PS score (0 or 1)	Ref	Ref	Ref	Ref
2	1.850	0.700 - 4.885	3.729	1.228 - 11.318
Unknown	0.609	0.232 - 1.600	1.089	0.360 - 3.295
COPD	0.867	0.416 - 0.957	0.728	0.396 - 1.338
Histologic type (non-squamous)	Ref	Ref	Ref	Ref
Squamous	0.869	0.555- 1.365	1.206	0.687 - 2.117
Stage (IIIA)	Ref	Ref	Ref	Ref
IIIB	1.639	1.014- 2.651	2.154	1.172 - 3.958
IIIC	1.980	1.008 – 3.889	1.522	0.684 - 3.385
History of surgery	0.614	–0.208 – 1.812	0.723	0.158 - 3.305
History of other cancer	2.091	–0.875 – 4.996	2.606	0.905 - 7.502
Patient age	1.000	0.972- 1.030	1.030	0.993 - 1.068
Gender	0.431	0.133 - 1.393	0.148	0.015 - 1.487
RT fraction	1.153	0.935- 1.421	0.934	0.752 - 1.161
RT dose in Gy	0.951	0.880 – 1.029	0.938	0.854 - 1.031

PFS: progression-free survival; OS: overall survival; HR: hazard ratio; CI: confidence interval; DC, durvalumab consolidation; BMI: body mass index; eGFR: estimated glomerular filtration rate; ECOG: Eastern Cooperative Oncology Group; PS: performance status; COPD: chronic obstructive pulmonary disease; RT, radiotherapy.
