# Supplementary figures and images for: A Propensity-Matched Retrospective Comparative Study with Historical Control to Determine the Real-World Effectiveness of Durvalumab after Concurrent Chemoradiotherapy in Unresectable Stage III Non-Small Cell Lung Cancer
Source: Cancers (Basel). 2023 Mar 5;15(5):1606. doi: 10.3390/cancers15051606 (PMC10000649; doi:10.3390/cancers15051606)

a

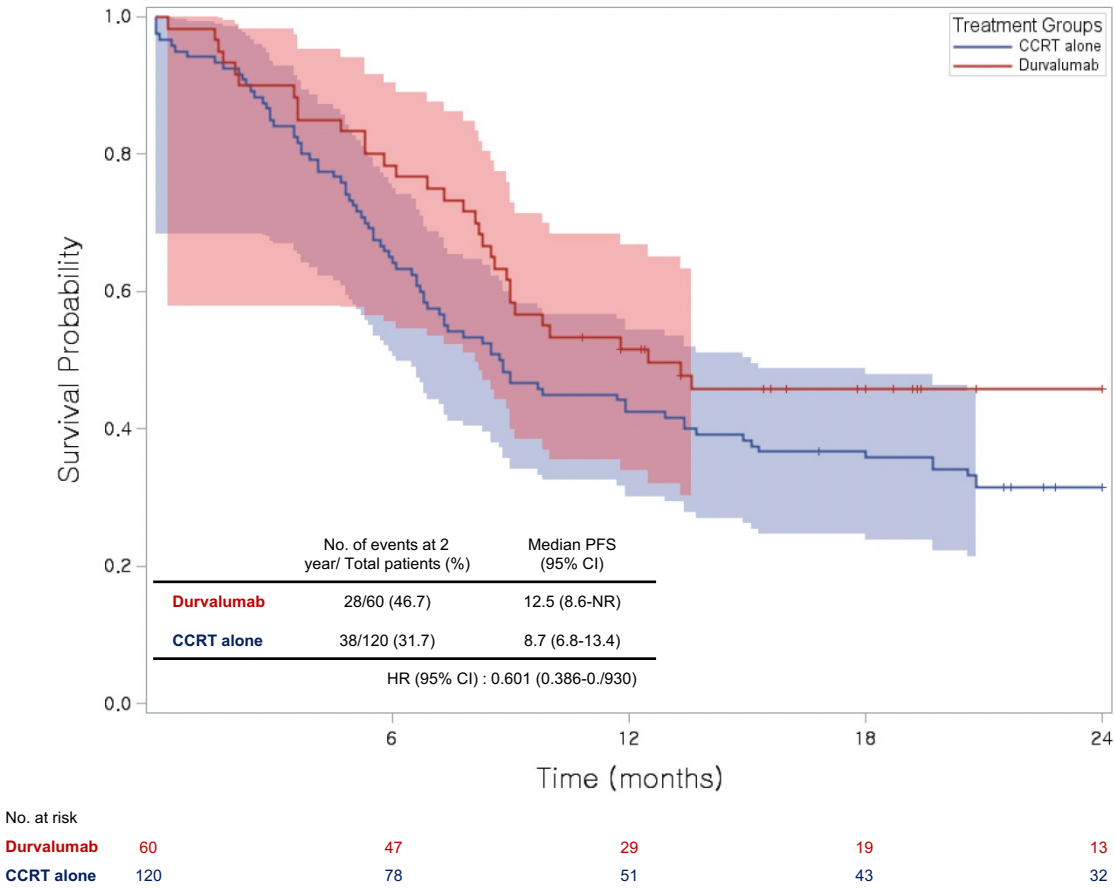

b

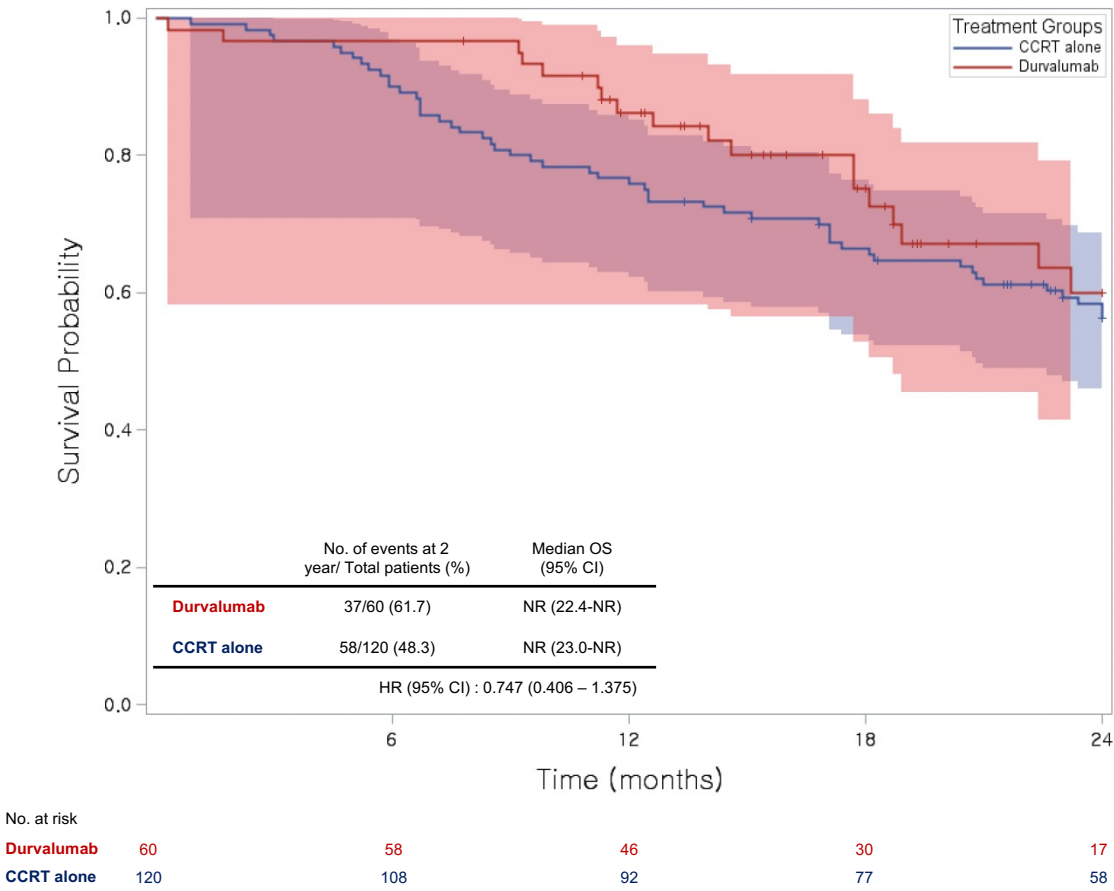

Supplement: Supplementary file 1 [file cancers-15-01606-s001.zip › Figure S1_1R.pdf]

a

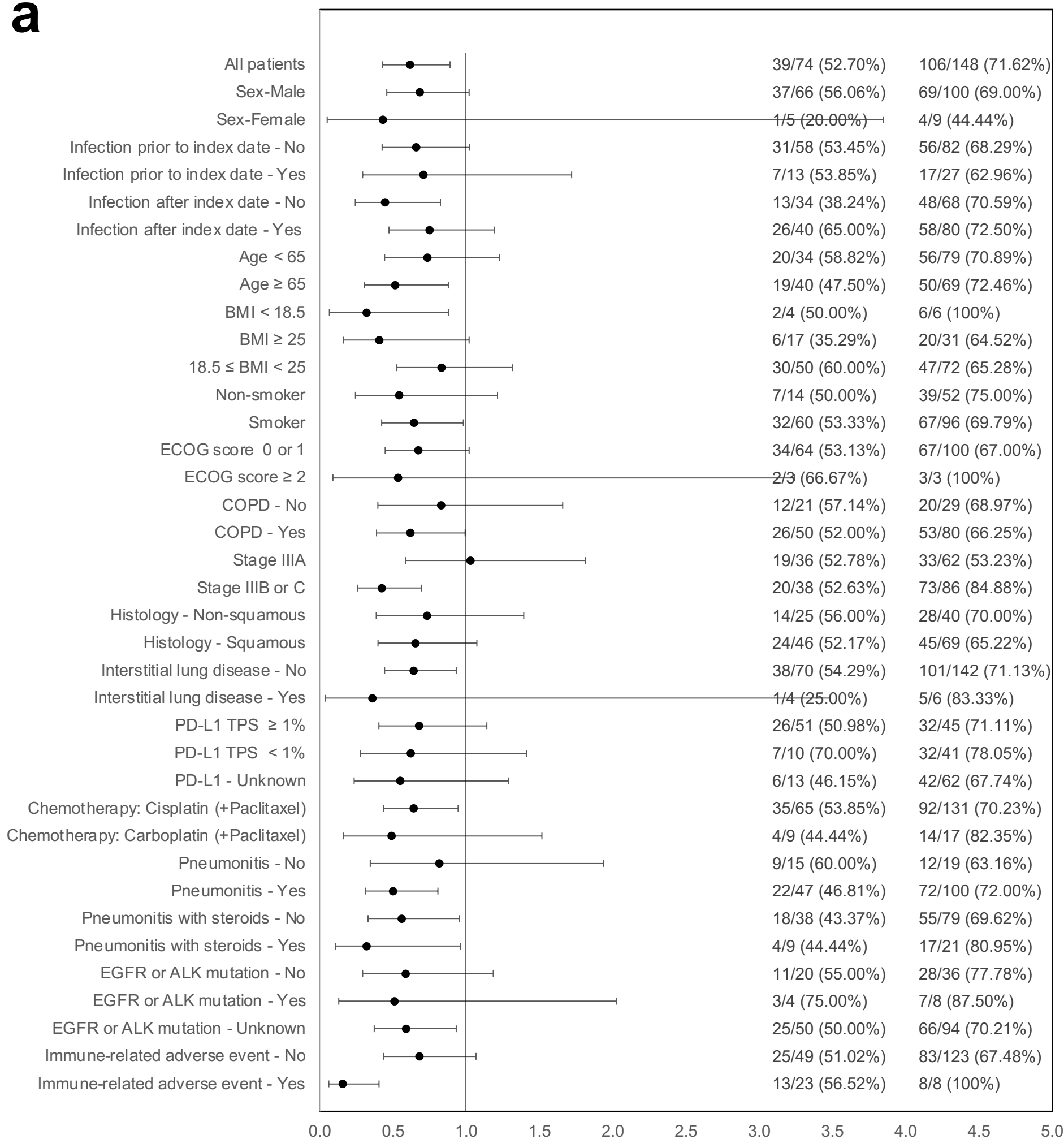

Supplement: Supplementary file 1 [file cancers-15-01606-s001.zip › Figure S2a.pdf]

b

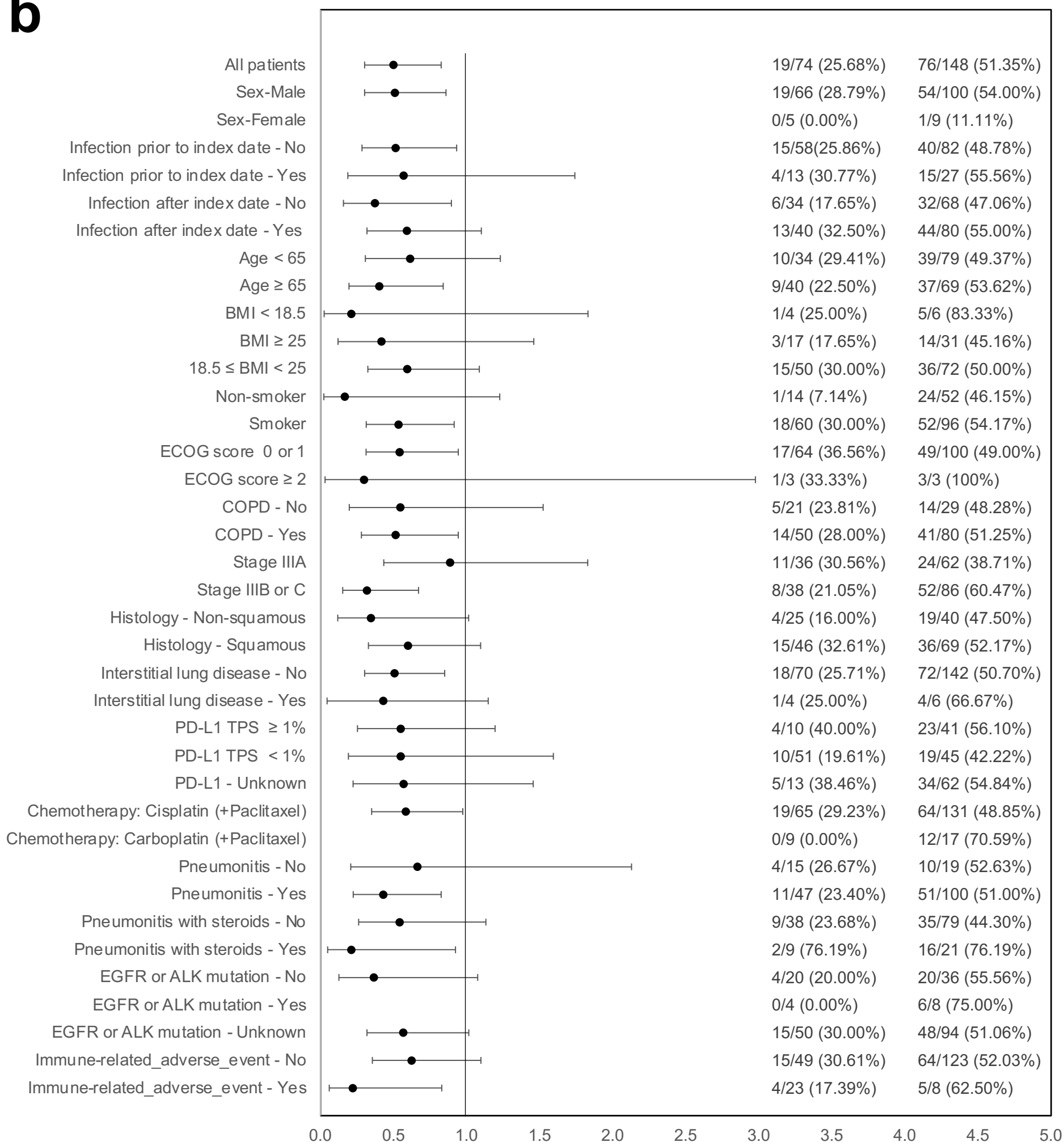

Supplement: Supplementary file 1 [file cancers-15-01606-s001.zip › Figure S2b.pdf]
